# Affinity-Enriched Plasma Proteomics for Biomarker Discovery in Abdominal Aortic Aneurysms

**DOI:** 10.3390/proteomes12040037

**Published:** 2024-12-09

**Authors:** Nicolai Bjødstrup Palstrøm, Kristian Boje Nielsen, Amanda Jessica Campbell, Mette Soerensen, Lars Melholt Rasmussen, Jes Sanddal Lindholt, Hans Christian Beck

**Affiliations:** 1 Center for Clinical Proteomics, Odense University Hospital, 5000 Odense, Denmark; kristian.boje.nielsen@rsyd.dk (K.B.N.); amanda.jessica.campbell@rsyd.dk (A.J.C.); hans.christian.beck@rsyd.dk (H.C.B.); 2 Department of Clinical Biochemistry, Odense University Hospital, 5000 Odense, Denmark; lars.melholt.rasmussen@rsyd.dk; 3 Research Unit for Epidemiology, Biostatistics and Biodemography, Department of Public Health, University of Southern Denmark, 5230 Odense, Denmark; msoerensen@health.sdu.dk; 4 Department of Cardiothoracic and Vascular Surgery, Odense University Hospital, 5000 Odense, Denmark; jes.sanddal.lindholt@rsyd.dk

**Keywords:** abdominal aortic aneurysm, affinity enrichment, mass spectrometry, machine learning, plasma biomarkers, proteomics

## Abstract

Abdominal aortic aneurysm (AAA) is a life-threatening condition characterized by the weakening and dilation of the abdominal aorta. Few diagnostic biomarkers have been proposed for this condition. We performed mass spectrometry-based proteomics analysis of affinity-enriched plasma from 45 patients with AAA and 45 matched controls to identify changes to the plasma proteome and potential diagnostic biomarkers. Gene ontology analysis revealed a significant upregulation of the proteins involved in inflammation, coagulation, and extracellular matrix in AAA patients, while proteins related to angiogenesis were among those downregulated. Using recursive feature elimination, we identified a subset of 10 significantly regulated proteins that were highly predictive of AAA. A random forest classifier trained on these proteins achieved an area under the curve (AUC) of 0.93 [95% CI: 0.91–0.95] using cross-validation. Further validation in a larger cohort is necessary to confirm these results.

## 1. Introduction

An abdominal aortic aneurysm (AAA) is a life-threatening vascular disorder characterized by a permanent and localized dilation of the abdominal aorta above >30 mm. AAAs result from the weakening of the abdominal aorta’s arterial wall, and while AAAs often remain asymptomatic for many years, sudden rupture of the aorta is possible. The rupture of an AAA is a medical emergency with high mortality rates, highlighting the need for early detection and monitoring [1]. 

The development of an AAA is a multifactorial process involving the interplay of genetics, lifestyle factors, and various cellular and molecular pathways. Specifically, the degradation of the extracellular matrix, inflammation, and loss of vascular smooth muscle cells (VSMC) have been implicated in the AAA pathogenesis [2,3]. Despite advances in treatments, the underlying mechanisms of AAA development and progression remain poorly understood, hindering the development of effective diagnostic and therapeutic strategies.

Analysis of clinically relevant samples is crucial for understanding the AAA’s pathophysiology. Previous studies have analyzed various clinical samples, including intraluminal thrombus (ILT) and arterial tissue, but plasma proteomics stands out for its potential in biomarker discovery [4,5]. For instance, studies using tandem mass tag (TMT) labeling and LC-MS/MS have previously identified differences in protein profiles among AAA patients and controls [6]. However, studies examining the potential biomarkers for AAA using mass spectrometry in plasma have been limited and have often been restricted to an analysis of small cohorts [6,7,8,9]. AAAs are often incidentally diagnosed by ultrasound or CT scans at unrelated examinations [10]. Although population screening programs have been implemented for certain at-risk groups in a few countries, broad implementation would not be practical due to various reasons, such as a reduced benefit of screening and limited availability of imaging resources [11,12,13]. In this context, plasma proteomics could offer a minimally invasive and cost-effective means for screening or monitoring disease progression. However, the vast dynamic range of plasma proteins and the presence of high-abundance proteins pose challenges, necessitating protein-enrichment strategies to enhance detection sensitivity [14].

Recent advances in protein-enrichment techniques have improved plasma proteomics, enabling the identification of novel biomarkers and providing deeper insights into many diseases. A previous head-to-head comparison of the multi-affinity removal system (MARS14) human 14 column, the proteominer protein equalization method, and four novel small-molecule affinity probes revealed that p-aminobenzamidine (ABA) was optimal for enriching low-abundant plasma proteins [15]. This current study introduces magnetic ABA affinity probes, adaptable to 96-well plate formats, for easier sample preparation. These probes were used to analyze plasma from AAA patients, identifying potential plasma protein biomarker candidates.

## 2. Materials and Methods

### 2.1. Study Population

This study was conducted using the plasma samples acquired from a randomized study, the Viborg Vascular (VIVA) screening trial, carried out in the Central Denmark Region, in which 25,078 Danish males between the ages of 65–74 years were invited to a screening for AAA, peripheral arterial disease, and hypertension between 2008 and 2011 [16]. All participants gave informed consent, and ethical approval was acquired from the Ethics Committee of Central Denmark Region (journal number: M-20080028). Participants who accepted the invitation were screened using an ultrasound scan of the abdominal aorta, and filled out a questionnaire providing information regarding age, height, weight, smoking, and previous history of cardiovascular disease. Diagnosis of an AAA was based on an anterior–posterior and inner–inner aortic diameter of at least 30 mm. An intraluminal thrombus (ILT) was determined to be present if there was a difference between the inner and outer areas of the aorta. Blood samples were drawn with consent for measurements of total cholesterol and C-reactive protein levels and for biobanking purposes. All blood samples were handled according to the hospital’s standard operating procedure for blood sampling, which included blood collection in 4 mL BD tubes containing 7.2 mg of K2-EDTA, followed by centrifugation at 2000× *g* for 10 min at room temperature. Plasma was separated into 1 mL aliquots into 1.3 mL Sarsted polypropylene tubes and stored at −80 °C immediately after venipuncture.

Baseline information regarding the usage of prescription drugs was acquired as part of the screening trial from the Danish National Prescription Register, which covers information regarding the prescription drugs purchased at Danish community pharmacies [17]. 

Using baseline data from the VIVA screening trial, a sub-cohort of 45 participants diagnosed with an AAA and 45 participants without an AAA for which plasma samples were available were selected. The participants in the two groups were deliberately selected to have no significant differences between them based on the acquired baseline data presented in Table 1, except for the presence of an AAA.

### 2.2. Preparation of Magnetic Affinity Probes

Tosylactivated superparamagnetic polystyrene beads (Dynabeads M-280, Invitrogen, catalog number: 14204, lot nr. 01181536) were coupled with ABA (4-aminobenzamidine dihydrochloride, Sigma-Aldrich, catalog number: 06880, lot MKCB0554V) according to the manufacturer’s instructions to create the magnetic affinity probes. Briefly, 100 µL of the beads was washed twice using 1000 µL of 0.1 M sodium borate buffer (pH 9.5), discarding the wash after each time. Then, 300 µL of ABA (300 µg ABA dissolved in 300 µL 0.1 M sodium borate) was incubated overnight with 200 µL of ammonium sulfate (3 M, pH 9.5) at room temperature. After incubation, magnetic beads coupled with ABA were washed thrice with 1000 µL of phosphate-buffered saline (PBS), discarding the wash after each time, and finally stored in 100 µL of PBS. 

### 2.3. Affinity Enrichment of Plasma Proteins

Enrichment of the plasma proteins using the magnetic affinity probes was performed by adding beads in a 5:1 ratio (sample/beads) with the plasma in a deep-well plate, along with 1000 µL of phosphate-buffered saline (PBS). After incubating the beads overnight at 5 °C on a rotary stand, a 96-well magnet was placed into the liquid to attract the magnetic beads to the surface of the magnet for 15 min. The magnetic beads were then transferred to a washing plate (Eppendorf 96-well/2000 µL of LoBind Deep-well plate, catalog number: 0030504305) pre-filled with 1250 µL of PBS. The washing procedure involved four repeated steps, each consisting of 15 min of washing on a rotary stand, followed by placing the 96-well magnet into the plate and allowing the magnetic beads to accumulate on the surface of the magnet for 15 min before transferring to a new washing plate. The magnet with the beads attached was then placed into a round-bottomed plate pre-filled with 40 µL of 0.2 M TEAB (triethylammonium bicarbonate). Proteins bound to the magnetic beads were then on-bead reduced using 5 mM DTT (dithiothreitol) at room temperature for 1 h, alkylated using 15 mM IAA (iodoacetamide) for 30 min at room temperature, and enzymatically digested using 0.25 µg of trypsin overnight at 37 °C using an orbital shaker. After digestion, placing a magnet on the bottom of the plate ensured that the magnetic beads were retained at the bottom, enabling the transfer of the digested proteins to a new plate. 

### 2.4. Isobaric Labeling Using TMTpro

Enriched plasma proteins were chemically labeled using 5 µg of TMTpro 16-plex in a 1:1 ratio (*w*/*w*) using the manufacturer’s standard procedure (Thermo Scientific, Rockford, IL, USA; catalog number: A44520, lot XB341490). TMTpro-tags 127N to 134N (15 TMTpro-tags) were randomly assigned to the different samples. A pool of all the digested samples was labeled with TMTpro-tag 126 and used as an internal standard to bridge the measurements between the six different TMTpro sets. 

Labeling efficiency was checked by analyzing a small sample of the pool by LC-MS/MS, searching the raw data with TMTpro as a variable modification, and calculating the proportion of labeled peptides in the data. Labeling efficiency exceeding 95% was deemed acceptable for further analysis. 

Prior to high pH fractionation, each set of labeled samples was pooled in equal amounts and purified using in-house made microcolumns packed with reversed-phase material (1:1, *w*/*w*, Poros R2, and Oligo R3).

### 2.5. High pH Fractionation of Labeled TMTpro Sets

Fractionation of the labeled and pooled TMTpro sets was performed virtually as previously described [18]. Briefly, fractionation was performed using a Dionex Ultimate 3000 RSLCNano system coupled to a Dionex 3000 Ultimate UV detector and a Dionex Ultimate 3000 autosampler configured as a fraction collector (Thermo Scientific, Bremen, Germany). The samples were loaded onto an ACQUITY C18 column (UPLC M-Class CSH, 130 Å, 1.7 µm bead size, 300 µm id × 100 mm length, Waters) and the peptides were separated into 11 fractions using a 25 min linear gradient from 10% buffer B (20 mM ammonium formate in 80% acetonitrile (CAN), pH 9.3) to 55% buffer B with a flow rate of 6 µL/min.

### 2.6. LC-MS Analysis

Nano-LC-MS/MS analysis of the samples was performed on an Orbitrap Exploris 480 mass spectrometer (Thermo Fisher Scientific, Bremen, Germany) equipped with a nano-HPLC interface (Dionex UltiMate 3000 nano-HPLC, Thermo Scientific, Bremen, Germany). Mass spectra were acquired using data-dependent acquisition (DDA) with an Orbitrap MS1 scan (400–1200 *m*/*z*, 120,000 resolution, 100% AGC, auto injection time) followed by an Orbitrap MS2 scan (30,000 resolution, TurboTMT enabled, HCD, 35% normalized collision energy, 0.7 m/z isolation width, 30 s dynamic exclusion, 200% AGC target, 86 milliseconds injection time). Field asymmetric waveform ion mobility spectrometry (FAIMS) was used with a standard resolution, 3.8 L/min gas flow, and two different compensation voltages (CV): −50 and −70. A cycle time of 1.5 s was allotted to each CV value. The samples were loaded on custom-made fused capillary pre-columns and separated on a custom-made C18 column using a linear gradient ranging from 90% to 86% solvent A (0.1% formic acid in water) to 30–34% B (0.1% formic acid in 80% acetonitrile) over 86 min followed by 4 min at 95% B and 4 min at 98% A at a flow rate of 250 nL/min.

### 2.7. Data Analysis

All 66 raw data files (11 fractions per TMTpro set) were processed using MSPepSearch and Sequest HT in parallel and integrated into Proteome Discoverer version 3.0 (Thermo Scientific, San Jose, CA, USA). All searches were performed against the SwissProt database restricted to humans (downloaded 23 January 2023, 20,330 entries) [19]. Sequest HT searches were performed using trypsin with two missed cleavages, an 8 ppm precursor mass tolerance, and a 0.05 Da fragment mass tolerance. Static modifications were limited to carbamidomethylation on cysteines and TMTpro on lysine and N-terminal amines, while methionine oxidation and deamidation of asparagine and glutamine were set as dynamic. A percolator was used for the FDR calculation (FDR 1%) and filtering of non-confident peptides. A single unique peptide was considered sufficient to indicate the presence of the intact protein if it also passed the FDR filter. Spectral library searches using MSPepSearch were used with a precursor mass tolerance and fragment mass tolerance of 15 ppm and our previously published TMTpro-specific spectral library [20].

### 2.8. Pathway Analysis

To obtain the basic functional information about proteins that were significantly regulated in relation to the presence of an AAA and to investigate potentially relevant biological pathways, ShinyGo (v. 0.8) was used to perform the gene set enrichment analysis based on the publically available Gene ontology (GO) [21,22]. Lists of upregulated and downregulated proteins were analyzed separately, and the settings were left at default. Proteins were ranked based on the fold enrichment as a measure of the strength of the enrichment, and Benjamini–Hochberg (FDR) was used to account for multiple testing (FDR < 0.05).

### 2.9. Statistical Analysis

For the descriptive statistics, categorical variables were summarized as counts with percentages, and for the continuous variables, means ± standard deviations, as well as medians with minimum and maximum values, were given. In cases where missing values were present for some variables, the number of participants for which the values were missing was given as a count with a percentage. For the categorical variables, a chi-squared test of independence was used to determine whether the frequencies of the distributions were statistically significant between the controls and those with an AAA. Similarly, for the continuous variables, a Student’s *t*-test was used to determine whether the difference in means between the two groups was statistically significant. For both analyses, a *p* < 0.05 was deemed statistically significant. 

The determination of differentially abundant proteins (DAPs) was based on a calculation of the fold-change and Student’s *t*-test. As *t*-tests were carried out for each individual protein, the resulting number of *t*-tests performed was quite large, which increased the probability of falsely deeming proteins to be significant. Therefore, a correction for multiple testing was performed using the Benjamini–Hochberg procedure for controlling the false discovery rate (FDR). A list of all proteins can be found in Supplementary Table S1.

Imputation of missing values in the proteomics data was performed prior to feature selection using a commonly applied imputation method based on the k-nearest neighbors (kNN) algorithm [18,23,24] using k = 10. It has previously been determined that imputation using kNN is reliable for TMT data with up to 50% missing values [25], and the same limit was therefore applied in this study. Feature selection was performed to identify a subset of proteins from the nominal statistically significant proteins, which could correctly separate participants with an AAA from those without. Recursive feature elimination (RFE) was used as the selection method, in which less important proteins are recursively removed until an optimal number of important proteins is retained. The ten most important proteins were then used to train a random forest (RF) classification model to identify participants with an AAA. The variable *mtry,* controlling the number of proteins randomly selected at each split in the decision tree, was not optimized during model training; instead, *mtry* = 2 was used. Robust estimation of model performance was estimated using 10-fold cross-validation with five repeats, and the receiver operating curve (ROC) and the associated area under the curve (AUC) were used as metrics to evaluate the model performance. The final performance estimate was based on the average across the repetitions. The computation of the confidence interval of the AUC is based on DeLong’s method and was computed based on 2000 stratified bootstrap replicates. All statistical analyses were carried out in R (v. 4.2.2) using the caret and randomForest packages for model training and the *pROC* package for model evaluation. Correlation analyses of the aortic diameter and protein abundance were carried out by calculating Pearson’s correlation coefficient for each individual protein and the aortic diameter using the cor.test-function in the *stats* package. A *p*-value was calculated for each correlation pair, but no attempt at correction for multiple testing was carried out. The R-code is publically available at https://nbpalstrom.github.io/ (accessed on 2 December 2024). 

## 3. Results

### 3.1. Patients and Study Design

The study cohort was selected from the Viborg Vascular (VIVA) screening trial, where participants were thoroughly characterized based on their initial measurements and questionnaires. To minimize the influence of confounding variables, which could potentially affect the expression of proteins, we purposely selected 45 patients with an AAA and 45 controls without an AAA, so none of the two groups of participants significantly differed in terms of their physiological variables (age, BMI, etc.), smoking status, total cholesterol, or the medications presented in Table 1. Based on the characteristics presented, the sole significant difference between the two groups of participants is the presence of an abdominal aortic aneurysm.

**Figure 1:**
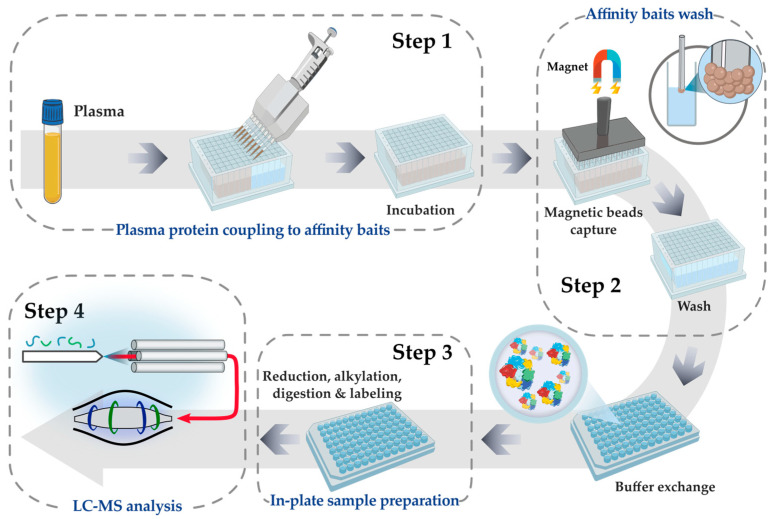
Affinity enrichment method overview. Plasma and magnetic affinity baits were incubated together in a deep-well plate, and the affinity baits captured and bound plasma protein during the incubation process. After incubation, using a magnet, the magnetic affinity baits were removed from the liquid, and the baits were then washed multiple times to remove unbound proteins and other contaminants. The plasma proteins, bound to affinity baits, were reduced, alkylated, and enzymatically digested prior to TMTpro labeling. The digested and labeled peptides were then analyzed using LC-MS.

We acquired plasma proteome profiles of all participants using mass spectrometry-based proteomic analysis of individual plasma samples. We utilized custom-made magnetic ABA affinity probes, as described in Section 2, for the enrichment of lower-abundant proteins, as previously published [15]. An overview of the proteomics workflow is depicted in Figure 1. 

**Table 1:** Descriptive statistics of study participants. For continuous variables, a Student’s *t*-test was performed to determine whether the difference in means for each group was statistically significant. For categorical variables, a chi-squared test of independence was performed to compare whether the frequencies of a categorical variable between the AAA patients and controls were statistically significant. *p* < 0.05 was deemed statistically significant *.

		Controls	AAA	*p*-Value *
		(*n* = 45)	(*n* = 45)	
Age (years)				
	- Mean (SD)	69.5 (2.82)	69.8 (2.57)	0.71
	- Median [Min, Max]	69.5 [65.4, 75.1]	69.6 [65.1, 75.0]	
Aortic diameter (mm)				
	- Mean (SD)	18.0 (2.05)	46.1 (11.6)	<0.01
	- Median [Min, Max]	18.0 [13.7, 22.7]	43.1 [33.7, 91.0]	
Body Mass Index (kg/m^2^)				
	- Mean (SD)	27.1 (1.46)	27.2 (1.68)	0.57
	- Median [Min, Max]	26.8 [25.2, 30.0]	27.2 [24.3, 30.0]	
Intraluminal thrombus		-	35 (77.8%)	
Systolic blood pressure				
	- Mean (SD)	149 (17.3)	151 (15.2)	0.50
	- Median [Min, Max]	146 [119, 203]	150 [121, 185]	
Preexisting conditions, *n* (%)				
Diabetes mellitus		5 (11.1%)	4 (8.9%)	0.97
	- Missing (%)	1 (2.2%)	0 (0%)	
Previous AMI		0 (0%)	2 (4.4%)	0.48
Previous angina		1 (2.2%)	2 (4.4%)	1.00
Previous CVD		3 (6.7%)	6 (13.3%)	0.48
Current smoking		45 (100%)	45 (100%)	1.00
Laboratory measurements				
C-reactive protein (mg/L)				
	- Mean (SD)	2.49 (2.12)	4.79 (10.1)	0.16
	- Median [Min, Max]	1.70 [0.10, 9.00]	2.60 [0.30, 66.10]	
	- Missing (%)	4 (8.9%)	4 (8.9%)	
Total Cholesterol (mmol/L)				
	- Mean (SD)	5.03 (1.09)	5.00 (0.87)	0.88
	- Median [Min, Max]	4.86 [2.00, 8.00]	4.83 [3.75, 7.11]	
Medications, *n* (%)				
ACE inhibitors		0 (0%)	0 (0%)	1.00
Angiotensin II inhibitors		3 (6.7%)	6 (13.3%)	0.48
Beta agonists		4 (8.9%)	2 (4.4%)	0.67
Beta blockers		9 (20.0%)	11 (24.4%)	0.80
Broncodilators		5 (11.1%)	3 (6.7%)	0.71
Calcium channel blockers		5 (11.1%)	8 (17.8%)	0.55
Clopidogrel		1 (2.2%)	5 (11.1%)	0.21
Magnyl		11 (24.4%)	19 (42.2%)	0.12
Statins		17 (37.8%)	23 (51.1%)	0.29
Warfarin		2 (4.4%)	1 (2.2%)	1.00

ACE: angiotensin-converting enzyme; AMI: acute myocardial infarct; CVD: cardiovascular disease; SD: standard deviation.

### 3.2. Impact of Abdominal Aortic Aneurysms on the Plasma Proteome

**Figure 2:**
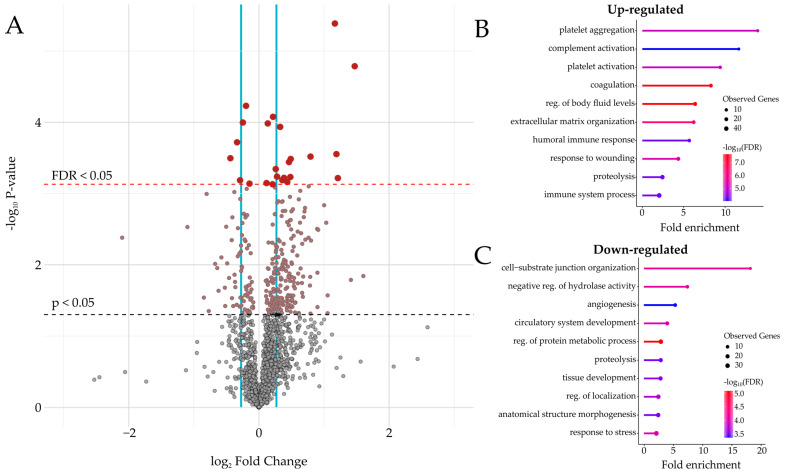
Differential plasma proteome alterations and functional annotations in abdominal aortic aneurysm. (**A**): Volcano plot showing the statistical significance versus fold change. The black dashed line indicates the unadjusted significance threshold, while the red dashed line indicates the threshold based on an FDR of 0.05. The blue lines indicate a fold-change of 0.83 and 1.2, respectively. (**B**): Gene enrichment analysis of the 211 upregulated proteins, and (**C**) 81 downregulated proteins in plasma samples from AAA individuals. The figure is limited to the ten most highly over-represented terms.

In total, 1610 quantifications for individual proteins were derived from the analysis of enriched plasma, and on average, 917 quantifications were achieved across the six TMTpro sets. Differential abundance analysis between the controls and AAA patients revealed 292 proteins to be significantly regulated, of which 24 proteins remained significantly regulated after multiple testing (FDR < 5%), accounting for 18% and 1.5% of the total quantified plasma proteome, respectively (Figure 2A, and Table 2). The complete list of differentially regulated proteins is available in Supplementary Table S1. The majority of the 292 regulated proteins were upregulated (72%; 211 proteins), while 28% (81 proteins) of the quantified proteins were downregulated. Of the 211 upregulated proteins, 156 exhibited a fold-change of >1.2 and a *p*-value of <0.05, with 13 of these proteins remaining significant after multiple testing. Similarly, among the 81 downregulated proteins, 32 proteins showed a fold-change of <0.83 and a *p*-value of <0.05, with three proteins also being significant after multiple tests. Grouping regulated proteins according to the functional annotation analysis revealed that approximately 25% of the upregulated proteins were associated with the immune system (Supplementary Table S2). Additionally, gene ontology terms related to platelets, complement activation, and coagulation were among the most highly over-represented terms related to the upregulated proteins (Figure 2B). Other related terms, including the organization of the extracellular matrix and response to wounding and proteolysis, showed a lesser degree of strength but were still highly significant. Conversely, the 81 downregulated proteins were largely related to response to stress (43%; 35 proteins), while cell-substrate junction organization, negative regulation of hydrolase activity, and angiogenesis were among the most over-represented categories for the downregulated proteins (Figure 2C, and Supplementary Table S3). 

### 3.3. Protein Prediction Model for Abdominal Aortic Aneurysm

**Figure 3:**
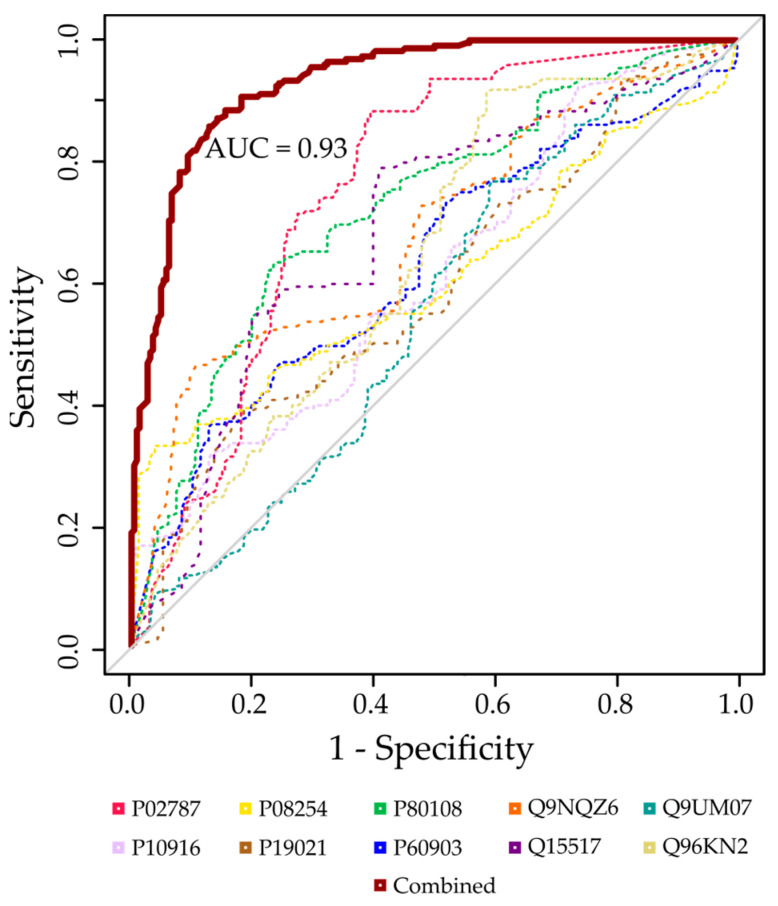
Prediction model based on plasma proteome for abdominal aortic aneurysm classification. The red line indicates the combined model with an area under the curve of 0.93 (95% CI: 0.91–0.95). Individual ROC curves for each protein included in the model with AUCs ranging from 0.55 to 0.75 are shown below the red line. P02787: Serotransferrin; P08254: Stromelysin-1; P80108: Phosphatidylinositol-glycan-specific phospholipase D; Q9NQZ6: Zinc finger C4H2 domain-containing protein; Q9UM07: Protein-arginine deiminase type-4; P10916: Myosin regulatory light chain 2; P19021: Peptidyl-glycine alpha-amidating monooxygenase; P60903: Protein S100-A10; Q15517: Corneodesmosin; Q96KN2: Carnosine dipeptidase 1.

Using the RFE feature selection algorithm, the ten most important proteins were selected from the significantly regulated proteins and used for the identification of AAA. These proteins either increased or decreased following the presence of an AAA. Among the ten proteins, only three were upregulated in AAA patients, while the remaining seven were downregulated. The random forest model, based on the panel of proteins, had a mean cross-validated AUC of 0.93 (95% confidence interval (CI): 0.91–0.95) for predicting an AAA with an associated sensitivity of 0.89 (SD = 0.155) and specificity of 0.84 (SD = 0.176) (Figure 3). To further investigate the importance of each protein, AUCs for each individual protein were calculated. The protein with the highest individual AUC was serotransferrin (AUC = 0.75), which was also the most important protein as determined by the feature selection algorithm. The remaining proteins had individual AUCs ranging from 0.55 (protein-arginine deiminase type-4) to 0.72 (Phosphatidylinositol-glycan-specific phospholipase D) (Table 3). Sensitivity analysis related to the presence of ILTs on the predictive performance revealed no substantial influence (Supplementary Material).

### 3.4. Correlation of Protein Abundance with Aortic Diameter

**Figure 4:**
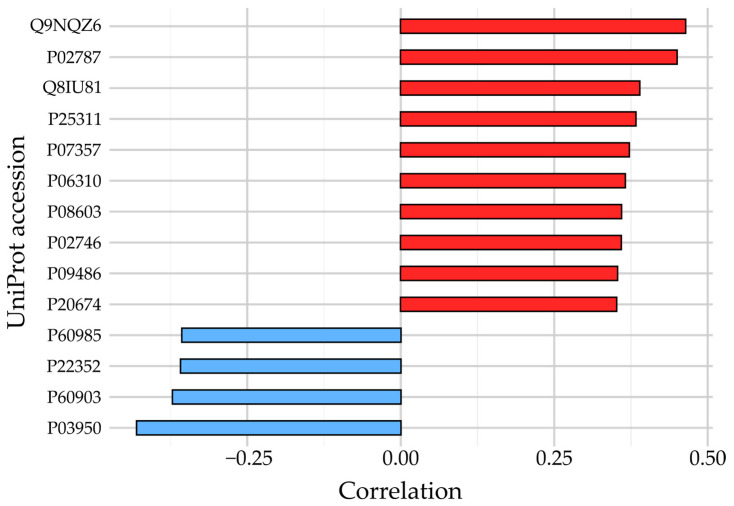
Pearson’s correlations between protein abundance and aortic diameter. The bar plot depicts selected protein correlations with aortic diameter for those proteins with an absolute correlation greater than 0.35. Red bars indicate positive correlations, while blue bars indicate negative correlations. Q9NQZ6: Zinc finger C4H2 domain-containing protein; P02787: Serotransferrin; Q8IU81: Interferon regulatory factor 2-binding protein 1; P25311: Zinc-alpha-2-glycoprotein; P07357: Complement component C8 alpha chain; P06310: Immunoglobulin kappa variable 2−30; P08603: Complement factor H; P02746: Complement C1q subcomponent subunit B; P09486: SPARC; P20674: Cytochrome c oxidase subunit 5A, mitochondrial; P60985: Keratinocyte differentiation-associated protein; P22352: Glutathione peroxidase 3; P60904: Protein S100-A10; P03950: Angiogenin.

The linear relationship between protein abundance and the aortic diameter was analyzed by Pearson’s correlation. In total, 211 proteins had significant correlations (*p* < 0.05) to the aortic diameter with Pearson correlation coefficients between −0.43 to 0.46. The strongest correlations are depicted in Figure 4, while a complete list of all 211 proteins with significant correlations is available in Supplementary Table S5. 

## 4. Discussion

In this study, we performed a comprehensive mass-spectrometry-based proteomic analysis of enriched plasma from patients with abdominal aortic aneurysms (AAA) and matched controls. The patients and matched controls were selected from a larger population-based screening cohort, which allowed us to match on far more clinically relevant parameters than previous studies. In terms of the patients with an AAA, large variations in the aortic diameter were observed with the cohort, including both small and very large aneurysms. Our findings revealed important biological processes that may contribute to AAA pathogenesis while also highlighting potential biomarkers that may prove relevant in terms of early diagnosis.

The identification of differentially abundant proteins revealed dysregulated biological processes that are biologically relevant in AAA pathogenesis. The functional annotation based on the gene ontology terms revealed that processes related to the complement system, extracellular matrix organization, and immune response were significantly altered. These findings are in line with previous studies, which associate inflammation and degradation of the extracellular matrix as central to the development and pathogenesis of AAA [26,27,28,29,30]. Proteins related to extracellular matrix organization included, among other things, different collagen types and several proteases, such as tolloid-like protein 1, matrix metalloproteinase-1, cathepsin G, and A disintegrin and metalloproteinase with thrombospondin motifs 5 (ADAMTS5). Matrix metalloproteinase-1 has previously been shown to be increased in AAA tissue [31] and associated with the rupture of an AAA [32]. Mast cells, which are the main source of cathepsin G, have previously been suggested to be involved in the pathogenesis of AAA, and a study in mice suggested that the absence of cathepsin G reduced the activity of elastin-degrading matrix metalloproteinases [33,34,35]. ADAMTS5 is a well-known enzyme involved in degrading the extracellular matrix and was found in a previous study to be differentially abundant in patients with aortic dissections, a condition that is also caused by a weakening of the extracellular matrix [36]. Previous murine studies have also suggested the involvement of ADAMTS5 in thoracic aortic aneurysm formation [37]. Additionally, cartilage oligomeric matrix protein, a protein that maintains the contractile phenotype of VSMCs [38] and CCN family member 2, associated with both VSMCs and AAAs [39,40], were also among the proteins related to the extracellular matrix organization. Among the downregulated proteins in the functional analysis were many proteins related to angiogenesis, the process of new blood vessel formation. Angiogenesis is thought to be involved in the development of the aneurysm, as well as the destructive processes within the vessel walls [33,41]. Numerous studies have linked angiogenesis and AAAs, and some studies have also investigated the effect of anti-angiogenic agents as a potential treatment strategy for AAAs in animal models [42,43,44,45,46,47]. This study appears to be the first to provide protein-level evidence for the proposed regulated pathways based on a comprehensive analysis of human plasma from patients with an AAA. The majority of previous research supporting the mentioned pathways in relation to AAAs has mainly relied on either genomics studies or single-target protein analysis, many of which were performed in animal models.

Our analyses in this study were influenced by previous plasma proteomics research of AAAs, but our current work provides a more refined analysis compared to previous studies by using more advanced proteomics techniques, as well as integrating machine learning to identify highly discriminative proteins. Earlier studies were primarily descriptive as well as limited in the number of patients included. One of the earliest studies compared the plasma profiles of 20 AAA patients and 20 matched controls, published in 2010, had isolated spots using two-dimensional electrophoresis gels and analyzed these spots with mass spectrometry [48]. The authors concluded that the limited number of proteins identified lacked the biological plausibility to be potential biomarkers for an AAA. Technological advancements since then, in terms of sample preparation, analytical techniques, and instrumentation, have meant that the quantification of several hundreds of proteins in plasma is no longer a dream but a reality. A more recent proteomics study of AAAs worth mentioning is by Henriksson et al., which analyzed TMT-labeled, fractionated plasma depleted of albumin and immunoglobulins from 12 patients with an AAA and eight matched controls using mass spectrometry and detected 522 proteins in total [6]. The authors identified eight proteins to be significantly regulated in AAA patients compared to controls. In comparison with our study, we quantified 1610 proteins in total and identified 292 proteins to be significantly regulated; among these were 24 proteins significant after adjusting for multiple testing None of the eight proteins identified by Henriksson et al. was among the 24 significant regulated proteins after multiple testing. Of the eight proteins identified by Henriksson et al. as potential biomarkers, two were not identified in our study, one (Hemoglobin subunit β) was found to be nominally significant (*p* < 0.05) but did not pass adjustment for multiple testing, and the remaining five were identified but not found to be significant in our study. Among the latter was C-reactive protein, which was specifically selected as a variable that should not differ between our case and control groups. This study design choice regarding our cohort likely contributed to the general lack of overlap between our study and Henriksson et al. Furthermore, the lack of overlap does not necessarily reflect the reliability of the proposed biomarkers of either study but instead highlights the challenges inherent to proteomics research. Differences related to sample types, such as the EDTA plasma being analyzed in our study, while Henriksson et al. analyzed citrate plasma, might limit the comparability, as it has previously been determined [49,50,51]. Additional factors, such as different experimental methodologies and statistical approaches between the two studies, will most definitely also have an impact. A comparison of quantified proteins from Henriksson et al. and the present study annotated with estimated protein abundances retrieved from PeptideAtlas revealed that our method allowed us to quantify 420 proteins at the sub-ng/mL level, while only 13 proteins quantified in Henriksson et al. could be quantified at this level (Supplementary Table S6).

Applying RFE feature selection, we narrowed down the 292 proteins to a subset of ten proteins that were most predictive of an AAA status. This approach, combined with random forest-based classification, enabled us to build a highly accurate model for distinguishing AAA patients from the controls, with an area under the curve of 0.93. Importantly, the robustness of the model, as demonstrated by cross-validation, supports the capability of these plasma proteins as potential biomarkers for an AAA. To our knowledge, this study is the first instance of attempting to classify an AAA using plasma proteomics; however, previous studies have attempted using single-protein measurement using other methods, such as an enzyme-linked immunosorbent assay, with varying results [52,53,54,55]. 

Several of the feature-selected proteins have previously been reported in relation to an AAA or pathways involved in AAA pathogenesis. Increased levels of serotransferrin, a protein responsible for iron transport, could potentially reflect an accumulation of iron in an intramural thrombus and the aneurysmal wall, which has previously been suggested to be involved in the pathology of AAA formation [28]. Additionally, increased levels of serotransferrin could also reflect a compensatory mechanism caused by anemia, a condition prevalent among AAA patients, in which the body attempts to increase iron transport in response to decreased levels of hemoglobin [56,57]. Carnosine dipeptidase 1, a member of the M20 metalloprotease family, has yet to be described in relation to AAAs but has previously been associated with an increased risk of nephropathy in type 2 diabetes patients [58,59]. Phosphatidylinositol-glycan-specific phospholipase D (GPLD1), an enzyme that hydrolyzes glycosylphosphatidylinositol (GPI) anchors on plasma membranes, has previously been found to be upregulated in plasma from AAA patients analyzed by mass spectrometry [60].GPLD1 plays an important role in inflammation due to GPLD1’s ability to hydrolyze the GPI anchors of several inflammatory membrane proteins, such as CD55 and CD59, which have previously been shown to be connected to AAAs [61,62]. GPLD1 is also responsible for the release of the urokinase plasminogen activator (uPA) receptor, which is a key component in the plasminogen activation system that is involved in aneurysmal degradation [63,64,65]. Stromelysin-1, also known as matrix metalloproteinase-3, is a known enzyme with specificity for fibronectin, laminin, and several collagens, as well as a known activator of plasminogen and matrix metalloproteinase-9, which is highly associated with AAAs [66,67,68]. It has previously been shown in mice that the deletion of MMP3 protects mice from developing an AAA, which suggests that MMP3 is especially important in relation to AAAs [69]. Protein S100-A10, a member of the S100 protein family, is an integral part of the cellular structural scaffolding, and through its binding with annexin-2, it interacts with several plasma membrane proteins [70]. S100A10 is also capable of cleaving plasminogen to plasmin and may account for up to 50% of the cellular plasmin generation [71,72]. There have not yet been any publications mentioning S100A10 in relation to AAAs, but several S100 proteins have previously been investigated in relation to AAAs [29,73]. Deficiency in protein-arginine deiminase type-4, a protein that catalyzes the citrullination of histones, thereby inhibiting the release of neutrophil extracellular traps, has been shown to reduce AAA formation in experimental models [74,75,76]. Peptidylglycine alpha-amidating monooxygenase, an essential protein in the biosynthesis of many neural and endocrine peptides and hormones, has previously been linked to the regulation of blood pressure through its interaction with atrial natriuretic peptides, as well as a predictor for different cardiovascular disease, including heart failure [77,78,79]. Neither zinc finger C4H2 domain-containing protein, corneodesmosin, or myosin regulatory light chain 2 had any previously demonstrated plausible association with AAAs.

The presence of an ILT present in many of the AAA cases included in this study could be considered a confounding effect that might influence the proteomic profiles of the proposed predictive proteins. The number of AAA cases with an ILT corresponded to 77.8% of cases, which translates well with the known distribution of 70–80% of cases in the literature [80]. To address the concern of ILTs affecting the results, a series of sensitivity analyses aimed at evaluating the influence of ILT on our results was performed. These included comparing the protein levels of the proteins included in the prediction models between ILT-positive and ILT-negative cases, and the controls. The additional tests revealed no discernable differences in the direction of the fold-change compared to the original dataset with the exception of serotransferrin, which its abundance was significantly higher in the ILT-positive cases compared to the ILT-negative cases. This change might highlight a potential influence of ILT. However, in the comparison of the ILT-positive and ILT-negative cases with controls, fold changes for all proteins included in the prediction model were consistent with the original analysis. This suggests that the presence of ILT does not affect the protein levels of predictive proteins in a substantial direction. Additional re-analyses of the random forest models included adding the ILT status as a predictor, as well as excluding ILT-positive cases from the prediction model. The analyses revealed that the addition of ILT status to the protein predictors only slightly improved the predictive performance of the model. Importantly, excluding ILT-positive cases from the prediction model did not diminish the model’s ability to distinguish between AAA patients and the controls in absolute numbers compared to the original model. However, we acknowledge that the number of ILT-negative cases was limited, resulting in a subgroup analysis that lacked the proper statistical power. While these sensitivity analyses suggest that the presence of ILT does not impair the ability of the identified protein panel to distinguish AAA patients from the controls, the potential influence of ILT cannot be fully ruled out, owing to the limited number of ILT-negative cases.

A limitation of this study is the lack of proteoform analysis. While our study provides valuable insights into the differential abundance of proteins in relation to the presence of an AAA, it does not capture the changes in the abundance of different proteoforms. As a result, our findings do not fully reflect the underlying patient-specific variations since these are not captured by the peptide-centric proteomic analyses used in this study. Future studies could incorporate proteoform characterization to better understand the clinical implications. Another limitation is the prolonged incubation time of our method, which might compromise the ability to detect proteins in their original state at the time of sampling, for example, due to residual proteolytic activity or a change in enzymatic or non-enzymatic post-translational modifications. An additional limitation is that no protease inhibitors were used during the sample preparation. We acknowledge that this might influence the proteome profile due to potential additional protease activity. Another potential limitation of our study is the presence of comorbidities (e.g., diabetes) and the use of certain medications (e.g., statin, ACE inhibitors, and antihypertensives) among the participants, as these factors are common in older populations and could theoretically influence the proteome. While we observed no statistically significant differences in these clinical variables, the existence of subtle biological effects related to these factors cannot fully be ruled out. Given the limited cohort size, additional subgroup analyses to test for the impact of these clinical variables were not feasible in this study. Future studies with larger cohorts should aim to further explore and adjust for the impact of comorbidities and medications on proteomic analysis in AAAs. Despite filtering criteria being applied to exclude weak spectra, based on the confidence scores and false discovery rate thresholds, and exclusion of proteins not measured in at least 50% of all participants, some lower-quality spectra could potentially have been retained, and we recognize that their presence may introduce variability. The current study lacks experimental validation of the proteins included in the prediction model. While our proteomic analysis provides valuable insights into potential biomarkers, it would be beneficial if future studies could incorporate additional experimental replicates or orthogonal validation to confirm the clinical relevance of these findings. In addition, the observational nature of this study is a limitation, specifically with regard to causality, as it restricts our ability to interpret the identified associations as potential biomarkers for abdominal aortic aneurysms.

## 5. Conclusions

This study demonstrates the potential of mass spectrometry-based proteomics to identify the plasma proteome alterations associated with biologically relevant pathways to an AAA. Combining protein changes with machine learning identified a biomarker panel with high diagnostic potential and biological plausibility. The identified biomarker candidates warrant further investigation into their specific roles in AAA development but hold promise for improving the early detection of an AAA, especially in areas where imaging is not available. Validation of these findings using more targeted approaches and in larger cohorts would be ideal next steps. 

## Figures and Tables

**Table 2:** Significantly regulated plasma proteins associated with the presence of abdominal aortic aneurysms. Proteins in plasma with significant differential abundance after adjusting for multiple testing (FDR < 0.05) in relation to the presence of AAA. Protein regulation was assessed by calculating the fold change, defined as the ratio of the mean protein abundance in AAA patients to the mean protein abundance in the control group. Missing values are presented as percentages of the total number of patients. An expanded table with additional details is presented in Supplementary Table S4.

UniProt Accession	Protein Name	Protein Regulation	Adjusted *p*-Value	Missing Values
Q9NQZ6	Zinc finger C4H2 domain-containing protein	2.25	0.0066	0.00
Q8IU81	Interferon regulatory factor 2-binding protein 1	2.77	0.0131	0.00
P02787	Serotransferrin	1.16	0.0267	0.00
P01023	Alpha-2-macroglobulin	1.10	0.0267	0.00
P07357	Complement component C8 alpha chain	1.25	0.0267	0.00
Q96KN2	Carnosine dipeptidase 1	0.87	0.0267	0.00
P03950	Angiogenin	0.84	0.0267	0.00
P60903	Protein S100-A10	0.79	0.0384	16.67
P05164	Myeloperoxidase	1.73	0.0439	0.00
P60985	Keratinocyte differentiation-associated protein	0.74	0.0439	0.00
P20594	Atrial natriuretic peptide receptor 2	1.40	0.0439	50.00
Q92918	Mitogen-activated protein kinase kinase kinase kinase 1	2.28	0.0439	66.67
P08709	Coagulation factor VII	1.38	0.0447	0.00
P11388	DNA topoisomerase 2-alpha	1.40	0.0496	0.00
P03956	Interstitial collagenase	1.31	0.0496	0.00
P06310	Immunoglobulin kappa variable 2–30	1.20	0.0496	0.00
P10643	Complement component C7	1.16	0.0496	0.00
P14174	Macrophage migration inhibitory factor	1.35	0.0496	0.00
P02746	Complement C1q subcomponent subunit B	1.21	0.0496	0.00
P08603	Complement factor H	1.08	0.0496	0.00
P04439	HLA class I histocompatibility antigen, A alpha chain	1.28	0.0496	0.00
P80108	Phosphatidylinositol-glycan-specific phospholipase D	0.90	0.0496	0.00
P22352	Glutathione peroxidase 3	0.82	0.0496	0.00
P38646	Stress-70 protein, mitochondrial	2.32	0.0496	83.33

**Table 3:** Proteins selected for the random forest prediction model. Proteins selected from the imputed significantly regulated proteins by the RFE algorithm as the optimal set of proteins to classify patients with an AAA. Proteins are sorted from highest to lowest importance according to the ranking made by the RFE algorithm.

UniProt Accession	Protein Name	AUC
P02787	Serotransferrin	0.75
Q96KN2	Carnosine dipeptidase 1	0.64
P80108	Phosphatidylinositol-glycan-specific phospholipase D	0.72
P08254	Stromelysin-1	0.60
Q9NQZ6	Zinc finger C4H2 domain-containing protein	0.68
P60903	Protein S100-A10	0.62
P19021	Peptidylglycine alpha-amidating monooxygenase	0.59
Q15517	Corneodesmosin	0.68
Q9UM07	Protein-arginine deiminase type-4	0.55
P10916	Myosin regulatory light chain 2	0.61

## Data Availability

The datasets generated and analyzed during the current study are not publicly available due to hospital guidelines and legislation regarding personal data. Data will be available from the corresponding author upon reasonable request and with permission of the Odense University Hospital Legal Department. R-code is available from https://nbpalstrom.github.io/ (2 December 2024).

## Supplementary Materials

The following supporting information can be downloaded at: https://www.mdpi.com/article/10.3390/proteomes12040037/s1, Table S1: Complete list of quantified proteins; Table S2: Gene Ontology analysis of nominal significantly up-regulated proteins; Table S3: Gene Ontology analysis of nominal significantly down-regulated proteins; Table S4: Expanded Table 2; Table S5: Protein correlations to aorta diameter; Table S6: Comparison of estimated plasma protein concentrations; Supplementary Material: Sensitivity analysis related to the presence of intraluminal thrombus (ILT) on the prediction of AAA.

## References

[1] Alasasfeh I., Abudawood R., Hwidi B.E., Al-Shami R. (2024). Ruptured abdominal aortic aneurysm discovered by pocket-sized ultrasound in a low resource setting: A case report. Int. J. Emerg. Med..

[2] Cho M.J., Lee M.R., Park J.G. (2023). Aortic aneurysms: Current pathogenesis and therapeutic targets. Exp. Mol. Med..

[3] Lu H., Du W., Ren L., Hamblin M.H., Becker R.C., Chen Y.E., Fan Y. (2021). Vascular Smooth Muscle Cells in Aortic Aneurysm: From Genetics to Mechanisms. J. Am. Heart Assoc..

[4] Behr Andersen C., Lindholt J.S., Urbonavicius S., Halekoh U., Jensen P.S., Stubbe J., Rasmussen L.M., Beck H.C. (2018). Abdominal Aortic Aneurysms Growth Is Associated with High Concentrations of Plasma Proteins in the Intraluminal Thrombus and Diseased Arterial Tissue. Arterioscler. Thromb. Vasc. Biol..

[5] Palstrom N.B., Matthiesen R., Rasmussen L.M., Beck H.C. (2022). Recent Developments in Clinical Plasma Proteomics-Applied to Cardiovascular Research. Biomedicines.

[6] Henriksson A.E., Lindqvist M., Sihlbom C., Bergstrom J., Bylund D. (2018). Identification of Potential Plasma Biomarkers for Abdominal Aortic Aneurysm Using Tandem Mass Tag Quantitative Proteomics. Proteomes.

[7] Spadaccio C., Di Domenico F., Perluigi M., Lusini M., Giorgi A., Schinina M.E., Blarzino C., Covino E., Chello M., Coccia R. (2012). Serum proteomics in patients with diagnosis of abdominal aortic aneurysm. Cardiovasc. Pathol..

[8] Gamberi T., Puglia M., Guidi F., Magherini F., Bini L., Marzocchini R., Modesti A., Modesti P.A. (2011). A proteomic approach to identify plasma proteins in patients with abdominal aortic aneurysm. Mol. Biosyst..

[9] Acosta-Martin A.E., Panchaud A., Chwastyniak M., Dupont A., Juthier F., Gautier C., Jude B., Amouyel P., Goodlett D.R., Pinet F. (2011). Quantitative mass spectrometry analysis using PAcIFIC for the identification of plasma diagnostic biomarkers for abdominal aortic aneurysm. PLoS ONE.

[10] Sevil F.C., Tort M., Ozer Gokaslan C., Sevil H., Becit N. (2022). Incidence, follow-up and outcomes of incidental abdominal aortic aneurysms in computed tomography. Interact. Cardiovasc. Thorac. Surg..

[11] Mansoor S.M., Rabben T., Hisdal J., Jorgensen J.J. (2023). Eleven-Year Outcomes of a Screening Project for Abdominal Aortic Aneurysm (AAA) in 65-Year-Old Men. Vasc. Health Risk Manag..

[12] Force U.S.P.S.T., Owens D.K., Davidson K.W., Krist A.H., Barry M.J., Cabana M., Caughey A.B., Doubeni C.A., Epling J.W., Kubik M. (2019). Screening for Abdominal Aortic Aneurysm: US Preventive Services Task Force Recommendation Statement. JAMA.

[13] Mell M.W. (2022). Challenges persist in screening for abdominal aortic aneurysms. J. Vasc. Surg..

[14] Anderson N.L., Anderson N.G. (2002). The human plasma proteome: History, character, and diagnostic prospects. Mol. Cell. Proteom..

[15] Palstrom N.B., Rasmussen L.M., Beck H.C. (2020). Affinity Capture Enrichment versus Affinity Depletion: A Comparison of Strategies for Increasing Coverage of Low-Abundant Human Plasma Proteins. Int. J. Mol. Sci..

[16] Lindholt J.S., Sogaard R. (2017). Population screening and intervention for vascular disease in Danish men (VIVA): A randomised controlled trial. Lancet.

[17] Pottegard A., Schmidt S.A.J., Wallach-Kildemoes H., Sorensen H.T., Hallas J., Schmidt M. (2017). Data Resource Profile: The Danish National Prescription Registry. Int. J. Epidemiol..

[18] Palstrom N.B., Overgaard M., Licht P., Beck H.C. (2023). Identification of Highly Sensitive Pleural Effusion Protein Biomarkers for Malignant Pleural Mesothelioma by Affinity-Based Quantitative Proteomics. Cancers.

[19] Magrane M., UniProt C. (2011). UniProt Knowledgebase: A hub of integrated protein data. Database.

[20] Palstrom N.B., Campbell A.J., Lindegaard C.A., Cakar S., Matthiesen R., Beck H.C. (2024). Spectral library search for improved TMTpro labelled peptide assignment in human plasma proteomics. Proteomics.

[21] Ge S.X., Jung D., Yao R. (2020). ShinyGO: A graphical gene-set enrichment tool for animals and plants. Bioinformatics.

[22] Ashburner M., Ball C.A., Blake J.A., Botstein D., Butler H., Cherry J.M., Davis A.P., Dolinski K., Dwight S.S., Eppig J.T. (2000). Gene ontology: Tool for the unification of biology. The Gene Ontology Consortium. Nat. Genet..

[23] Jadhav A., Pramod D., Ramanathan K. (2019). Comparison of Performance of Data Imputation Methods for Numeric Dataset. Appl. Artif. Intell..

[24] Harris L., Fondrie W.E., Oh S., Noble W.S. (2023). Evaluating Proteomics Imputation Methods with Improved Criteria. J. Proteome Res..

[25] Palstrom N.B., Matthiesen R., Beck H.C. (2020). Data Imputation in Merged Isobaric Labeling-Based Relative Quantification Datasets. Methods Mol. Biol..

[26] Lindholt J.S., Shi G.P. (2006). Chronic inflammation, immune response, and infection in abdominal aortic aneurysms. Eur. J. Vasc. Endovasc. Surg..

[27] Liu Z., Morgan S., Ren J., Wang Q., Annis D.S., Mosher D.F., Zhang J., Sorenson C.M., Sheibani N., Liu B. (2015). Thrombospondin-1 (TSP1) contributes to the development of vascular inflammation by regulating monocytic cell motility in mouse models of abdominal aortic aneurysm. Circ. Res..

[28] Sawada H., Hao H., Naito Y., Oboshi M., Hirotani S., Mitsuno M., Miyamoto Y., Hirota S., Masuyama T. (2015). Aortic iron overload with oxidative stress and inflammation in human and murine abdominal aortic aneurysm. Arterioscler. Thromb. Vasc. Biol..

[29] Yang H., Zhou T., Stranz A., DeRoo E., Liu B. (2021). Single-Cell RNA Sequencing Reveals Heterogeneity of Vascular Cells in Early Stage Murine Abdominal Aortic Aneurysm-Brief Report. Arterioscler. Thromb. Vasc. Biol..

[30] Li T., Jiang B., Li X., Sun H.Y., Li X.T., Jing J.J., Yang J. (2018). Serum matrix metalloproteinase-9 is a valuable biomarker for identification of abdominal and thoracic aortic aneurysm: A case-control study. BMC Cardiovasc. Disord..

[31] Annabi B., Shedid D., Ghosn P., Kenigsberg R.L., Desrosiers R.R., Bojanowski M.W., Beaulieu E., Nassif E., Moumdjian R., Beliveau R. (2002). Differential regulation of matrix metalloproteinase activities in abdominal aortic aneurysms. J. Vasc. Surg..

[32] Wilson W.R., Anderton M., Choke E.C., Dawson J., Loftus I.M., Thompson M.M. (2008). Elevated plasma MMP1 and MMP9 are associated with abdominal aortic aneurysm rupture. Eur. J. Vasc. Endovasc. Surg..

[33] Swedenborg J., Mayranpaa M.I., Kovanen P.T. (2011). Mast cells: Important players in the orchestrated pathogenesis of abdominal aortic aneurysms. Arterioscler. Thromb. Vasc. Biol..

[34] Mayranpaa M.I., Trosien J.A., Fontaine V., Folkesson M., Kazi M., Eriksson P., Swedenborg J., Hedin U. (2009). Mast cells associate with neovessels in the media and adventitia of abdominal aortic aneurysms. J. Vasc. Surg..

[35] Wang J., Sukhova G.K., Liu J., Ozaki K., Lesner A., Libby P., Kovanen P.T., Shi G.P. (2015). Cathepsin G deficiency reduces periaortic calcium chloride injury-induced abdominal aortic aneurysms in mice. J. Vasc. Surg..

[36] Zeng T., Gan J., Liu Y., Shi L., Lu Z., Xue Y., Xiong R., Liu L., Yang Z., Lin Y. (2020). ADAMTS-5 Decreases in Aortas and Plasma From Aortic Dissection Patients and Alleviates Angiotensin II-Induced Smooth Muscle-Cell Apoptosis. Front. Cardiovasc. Med..

[37] Fava M., Barallobre-Barreiro J., Mayr U., Lu R., Didangelos A., Baig F., Lynch M., Catibog N., Joshi A., Barwari T. (2018). Role of ADAMTS-5 in Aortic Dilatation and Extracellular Matrix Remodeling. Arterioscler. Thromb. Vasc. Biol..

[38] Jia Y., Wang M., Mao C., Yu F., Wang Y., Xiao R., Jiang C., Zheng L., Xu Q., Zheng M. (2018). COMP-prohibitin 2 interaction maintains mitochondrial homeostasis and controls smooth muscle cell identity. Cell Death Dis..

[39] Larsen J.H., Rasmussen L.M., Lindholt J.S., Steffensen L.B. (2019). Plasma CCN2 is independently related to subsequent need for abdominal aorta aneurysm repair. Growth Factors.

[40] Wang Y., Liu X., Xu Q., Xu W., Zhou X., Leask A., Lin Z. (2023). CCN2 deficiency in smooth muscle cells triggers cell reprogramming and aggravates aneurysm development. JCI Insight.

[41] Thompson M.M., Jones L., Nasim A., Sayers R.D., Bell P.R. (1996). Angiogenesis in abdominal aortic aneurysms. Eur. J. Vasc. Endovasc. Surg..

[42] Zalewski D., Chmiel P., Kolodziej P., Borowski G., Feldo M., Kocki J., Bogucka-Kocka A. (2023). Dysregulations of Key Regulators of Angiogenesis and Inflammation in Abdominal Aortic Aneurysm. Int. J. Mol. Sci..

[43] Paik D.C., Fu C., Bhattacharya J., Tilson M.D. (2004). Ongoing angiogenesis in blood vessels of the abdominal aortic aneurysm. Exp. Mol. Med..

[44] Sano M., Sasaki T., Hirakawa S., Sakabe J., Ogawa M., Baba S., Zaima N., Tanaka H., Inuzuka K., Yamamoto N. (2014). Lymphangiogenesis and angiogenesis in abdominal aortic aneurysm. PLoS ONE.

[45] Zhang M., Sui W., Cheng C., Xue F., Tian Z., Cheng J., Zhang J., Zhang T., Zhang J., Wang W. (2021). Erythropoietin promotes abdominal aortic aneurysms in mice through angiogenesis and inflammatory infiltration. Sci. Transl. Med..

[46] Escudero P., Navarro A., Ferrando C., Furio E., Gonzalez-Navarro H., Juez M., Sanz M.J., Piqueras L. (2015). Combined treatment with bexarotene and rosuvastatin reduces angiotensin-II-induced abdominal aortic aneurysm in apoE(-/-) mice and angiogenesis. Br. J. Pharmacol..

[47] Miwa K., Nakashima H., Aoki M., Miyake T., Kawasaki T., Iwai M., Oishi M., Kataoka K., Ohgi S., Ogihara T. (2005). Inhibition of ets, an essential transcription factor for angiogenesis, to prevent the development of abdominal aortic aneurysm in a rat model. Gene Ther..

[48] Nordon I.M., Brar R., Hinchliffe R.J., Cockerill G., Thompson M.M. (2010). Proteomics and pitfalls in the search for potential biomarkers of abdominal aortic aneurysms. Vascular.

[49] Smit N.P.M., Romijn F., van Ham V.J.J., Reijnders E., Cobbaert C.M., Ruhaak L.R. (2023). Quantitative protein mass-spectrometry requires a standardized pre-analytical phase. Clin. Chem. Lab. Med..

[50] Ilies M., Iuga C.A., Loghin F., Dhople V.M., Thiele T., Volker U., Hammer E. (2017). Impact of blood sample collection methods on blood protein profiling studies. Clin. Chim. Acta.

[51] Geyer P.E., Voytik E., Treit P.V., Doll S., Kleinhempel A., Niu L., Muller J.B., Buchholtz M.L., Bader J.M., Teupser D. (2019). Plasma Proteome Profiling to detect and avoid sample-related biases in biomarker studies. EMBO Mol. Med..

[52] Wu J., Wang W., Xie T., Chen Z., Zhou L., Song X., Kan H., Lv Y., Wu L., Li F. (2022). Identification of Novel Plasma Biomarkers for Abdominal Aortic Aneurysm by Protein Array Analysis. Biomolecules.

[53] Xie T., Yin L., Guo D., Zhang Z., Chen Y., Liu B., Wang W., Zheng Y. (2021). The potential role of plasma fibroblast growth factor 21 as a diagnostic biomarker for abdominal aortic aneurysm presence and development. Life Sci..

[54] Molacek J., Treska V., Zeithaml J., Hollan I., Topolcan O., Pecen L., Slouka D., Karlikova M., Kucera R. (2019). Blood biomarker panel recommended for personalized prediction, prognosis, and prevention of complications associated with abdominal aortic aneurysm. EPMA J..

[55] Klopf J., Demyanets S., Brekalo M., Eilenberg W., Wojta J., Neumayer C., Brostjan C., Stojkovic S. (2022). Soluble ST2 as a Potential Biomarker for Abdominal Aortic Aneurysms-A Single-Center Retrospective Cohort Study. Int. J. Mol. Sci..

[56] Diehm N., Benenati J.F., Becker G.J., Quesada R., Tsoukas A.I., Katzen B.T., Kovacs M. (2007). Anemia is associated with abdominal aortic aneurysm (AAA) size and decreased long-term survival after endovascular AAA repair. J. Vasc. Surg..

[57] Ogun A.S., Adeyinka A. (2024). Biochemistry, Transferrin. StatPearls.

[58] Chakkera H.A., Hanson R.L., Kobes S., Millis M.P., Nelson R.G., Knowler W.C., Distefano J.K. (2011). Association of variants in the carnosine peptidase 1 gene (CNDP1) with diabetic nephropathy in American Indians. Mol. Genet. Metab..

[59] Ahluwalia T.S., Lindholm E., Groop L.C. (2011). Common variants in CNDP1 and CNDP2, and risk of nephropathy in type 2 diabetes. Diabetologia.

[60] Wallinder J., Bergstrom J., Henriksson A.E. (2012). Discovery of a novel circulating biomarker in patients with abdominal aortic aneurysm: A pilot study using a proteomic approach. Clin. Transl. Sci..

[61] Hinterseher I., Erdman R., Donoso L.A., Vrabec T.R., Schworer C.M., Lillvis J.H., Boddy A.M., Derr K., Golden A., Bowen W.D. (2011). Role of complement cascade in abdominal aortic aneurysms. Arterioscler. Thromb. Vasc. Biol..

[62] Wu G., Chen T., Shahsafaei A., Hu W., Bronson R.T., Shi G.P., Halperin J.A., Aktas H., Qin X. (2010). Complement regulator CD59 protects against angiotensin II-induced abdominal aortic aneurysms in mice. Circulation.

[63] Nessvi Otterhag S., Gottsater A., Acosta S., Palmqvist B., Lindblad B. (2014). Inflammatory mediators after endovascular aortic aneurysm repair. Cytokine.

[64] Moller L.B. (1993). Structure and function of the urokinase receptor. Blood Coagul. Fibrinolysis.

[65] Lindholt J.S., Jorgensen B., Shi G.P., Henneberg E.W. (2003). Relationships between activators and inhibitors of plasminogen, and the progression of small abdominal aortic aneurysms. Eur. J. Vasc. Endovasc. Surg..

[66] Arza B., Hoylaerts M.F., Felez J., Collen D., Lijnen H.R. (2000). Prostromelysin-1 (proMMP-3) stimulates plasminogen activation by tissue-type plasminogen activator. Eur. J. Biochem..

[67] Ogata Y., Enghild J.J., Nagase H. (1992). Matrix metalloproteinase 3 (stromelysin) activates the precursor for the human matrix metalloproteinase 9. J. Biol. Chem..

[68] Sakalihasan N., Delvenne P., Nusgens B.V., Limet R., Lapiere C.M. (1996). Activated forms of MMP2 and MMP9 in abdominal aortic aneurysms. J. Vasc. Surg..

[69] Hadi T., Boytard L., Silvestro M., Alebrahim D., Jacob S., Feinstein J., Barone K., Spiro W., Hutchison S., Simon R. (2018). Macrophage-derived netrin-1 promotes abdominal aortic aneurysm formation by activating MMP3 in vascular smooth muscle cells. Nat. Commun..

[70] Okura G.C., Bharadwaj A.G., Waisman D.M. (2023). Recent Advances in Molecular and Cellular Functions of S100A10. Biomolecules.

[71] Miller V.A., Madureira P.A., Kamaludin A.A., Komar J., Sharma V., Sahni G., Thelwell C., Longstaff C., Waisman D.M. (2017). Mechanism of plasmin generation by S100A10. Thromb. Haemost..

[72] Madureira P.A., O’Connell P.A., Surette A.P., Miller V.A., Waisman D.M. (2012). The biochemistry and regulation of S100A10: A multifunctional plasminogen receptor involved in oncogenesis. J. Biomed. Biotechnol..

[73] Chen S., Yang D., Liu B., Chen Y., Ye W., Chen M., Zheng Y. (2021). Identification of crucial genes mediating abdominal aortic aneurysm pathogenesis based on gene expression profiling of perivascular adipose tissue by WGCNA. Ann. Transl. Med..

[74] Ibrahim N., Bleichert S., Klopf J., Kurzreiter G., Hayden H., Knobl V., Artner T., Krall M., Stiglbauer-Tscholakoff A., Oehler R. (2024). Reducing Abdominal Aortic Aneurysm Progression by Blocking Neutrophil Extracellular Traps Depends on Thrombus Formation. JACC Basic. Transl. Sci..

[75] Wei M., Wang X., Song Y., Zhu D., Qi D., Jiao S., Xie G., Liu Y., Yu B., Du J. (2021). Inhibition of Peptidyl Arginine Deiminase 4-Dependent Neutrophil Extracellular Trap Formation Reduces Angiotensin II-Induced Abdominal Aortic Aneurysm Rupture in Mice. Front. Cardiovasc. Med..

[76] Yang S., Chen L., Wang Z., Chen J., Ni Q., Guo X., Liu W., Lv L., Xue G. (2023). Neutrophil extracellular traps induce abdominal aortic aneurysm formation by promoting the synthetic and proinflammatory smooth muscle cell phenotype via Hippo-YAP pathway. Transl. Res..

[77] O’Donnell P.J., Driscoll W.J., Back N., Muth E., Mueller G.P. (2003). Peptidylglycine-alpha-amidating monooxygenase and pro-atrial natriuretic peptide constitute the major membrane-associated proteins of rat atrial secretory granules. J. Mol. Cell. Cardiol..

[78] Yoo H.J., Kim M., Kim M., Chae J.S., Lee S.H., Lee J.H. (2017). The peptidylglycine-alpha-amidating monooxygenase (PAM) gene rs13175330 A>G polymorphism is associated with hypertension in a Korean population. Hum. Genom..

[79] Kaufmann P., Bergmann A., Melander O. (2021). Novel insights into peptide amidation and amidating activity in the human circulation. Sci. Rep..

[80] Piechota-Polanczyk A., Jozkowicz A., Nowak W., Eilenberg W., Neumayer C., Malinski T., Huk I., Brostjan C. (2015). The Abdominal Aortic Aneurysm and Intraluminal Thrombus: Current Concepts of Development and Treatment. Front. Cardiovasc. Med..

