# Supplementary material for: Affinity-Enriched Plasma Proteomics for Biomarker Discovery in Abdominal Aortic Aneurysms
Source: Proteomes. 2024 Dec 9;12(4):37. doi: 10.3390/proteomes12040037 (PMC11678615; doi:10.3390/proteomes12040037)
Supplement: Supplementary file 1 [file proteomes-12-00037-s001.zip › Supplementary Material.pdf]

Supplementary Material: Sensitivity analysis related to the presence of intraluminal thrombus (ILT) on the prediction of AAA

The intraluminal thrombus (ILT) present in many cases of abdominal aortic aneurysms (AAA) in this study could be a potential confounding variable in the prediction of AAA. Since an ILT might influence the plasma protein levels, it is possible that the presence of an ILT will also affect the predictive capability of the proposed protein panel. To address this concern, we performed a series of sensitivity analyses to evaluate the influence of ILT on the protein regulation and predictive power of our Random Forest model. The steps taken are summarized below:

1. Annotation of ILT status:
    a. Information about the inner and outer area of the aorta was recorded for each AAA patient.
    b. In cases where there were a difference between these two areas was determined to be indicative of the presence of an ILT.
2. Protein-level comparisons:
    a. Additional t-tests for the proteins included in the prediction model were made for the following comparisons:
        i. ILT-positive vs. ILT-negative cases
        ii. ILT-positive cases vs. controls
        iii. ILT-negative cases vs. controls
    b. This analysis was aimed to evaluate whether the presence of an ILT would influence the protein abundance and associated fold-changes of the protein included in the model
3. Re-analysis of the Random Forest models:
    a. The Random Forest prediction models were re-analyzed under two additional scenarios:
        i. Including ILT status as an additional prediction variable in the original model
        ii. Excluding ILT-positive cases entirely from the original model to asses the model's performance in separating AAA-patients (without ILTs) and controls

Summary of findings:

- Protein-level comparisons:
    - Proteins included in the prediction model showed consistent fold-change directions to the original data – except for serotransferrin, which was significantly increased in ILT-positive cases compared to ILT-negative cases.
    - Comparisons with the controls suggest that regardless of ILT status, the fold-change direction remain in the same direction as the original dataset. P-values are excluded from the comparisons since these are influenced by the number of cases.

- Re-analysis of the Random Forest models:
    - Including ILT status as a variable in the prediction model slightly improved the performance of the model, but the change was not substantial.
    - Excluding ILT-positive cases did not diminish the model ability to accurately predict AAA-patients and controls in absolute values. However, the limited number of ILT-negative cases did constrain the statistical power.

While this series of sensitivity analyses support that ILT status does not substantially influence the identified protein panel's ability to distinguish AAA patients and controls, we acknowledge that further validation in larger, more balanced cohort of ILT-status would be beneficial to assess the impact of ILTs.

Table 1. *Comparisons of protein level related to the presence of intraluminal thrombus (ILT). Adjustment for multiple testing was not taken into consideration.*

		Original		ILT+/ILT-		ILT+/Ctrl		ILT-/Ctrl	
UniProt accession	Protein name	Fold change	p-value	Fold change	p-value	Fold change	p-value	Fold change	p-value
P02787	Serotransferrin	1.16	8.37E-05	1.09	1.07E-02	1.18	3.82E-05	1.09	3.60E-02
Q96KN2	Carnosine dipeptidase 1	0.87	5.87E-05	0.96	3.02E-01	0.86	9.87E-05	0.90	1.54E-02
P80108	Phosphatidylinositol-glycan-specific phospholipase D	0.90	7.25E-04	0.96	4.10E-01	0.90	8.54E-04	0.93	9.23E-02
P08254	Stromelysin-1	0.88	2.23E-02	1.04	6.18E-01	0.88	5.53E-02	0.85	2.89E-02
Q9NQZ6	Zinc finger C4H2 domain-containing protein	2.25	4.10E-06	1.25	3.10E-01	2.35	1.95E-05	1.88	4.94E-02
P60903	Protein S100-A10	0.79	1.91E-04	1.03	6.42E-01	0.82	1.12E-03	0.80	6.73E-04
P19021	Peptidylglycine alpha-amidating monooxygenase	0.71	9.54E-03	1.13	4.12E-01	0.78	3.15E-02	0.69	1.10E-02
Q15517	Corneodesmosin	0.82	1.19E-03	1.00	9.76E-01	0.82	1.21E-03	0.82	9.58E-02
Q9UM07	Protein-arginine deiminase type-4	1.22	3.74E-02	0.99	9.40E-01	1.21	3.58E-02	1.22	6.46E-02
P10916	Myosin regulatory light chain 2	0.83	3.72E-02	0.92	2.94E-01	0.82	2.81E-02	0.88	2.03E-01

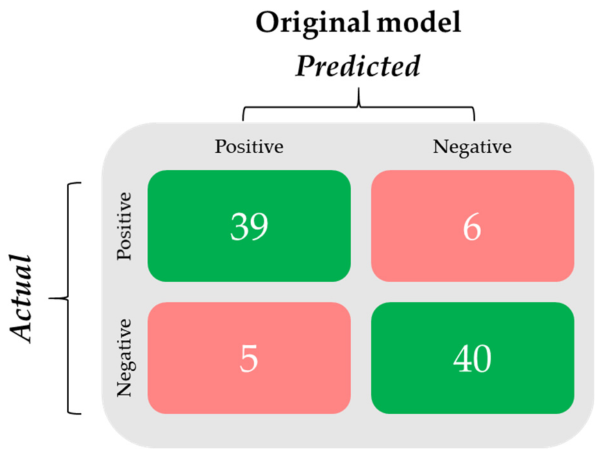

Figure 1. *Confusion matrix demonstrating the predictive performance of the original prediction model.*

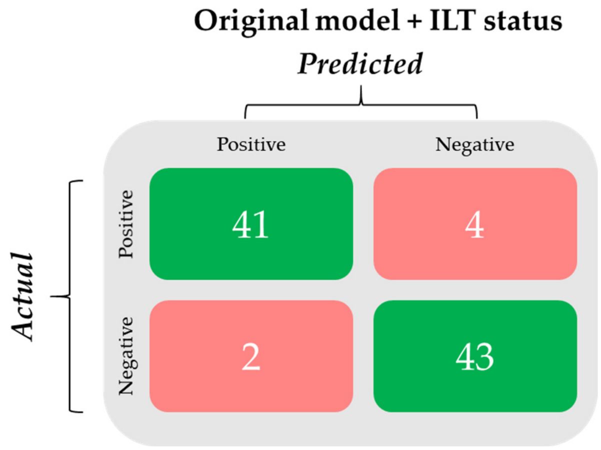

Figure 2. *Confusion matrix demonstrating the predictive performance of the original prediction model with ILT included as a predictive variable.*

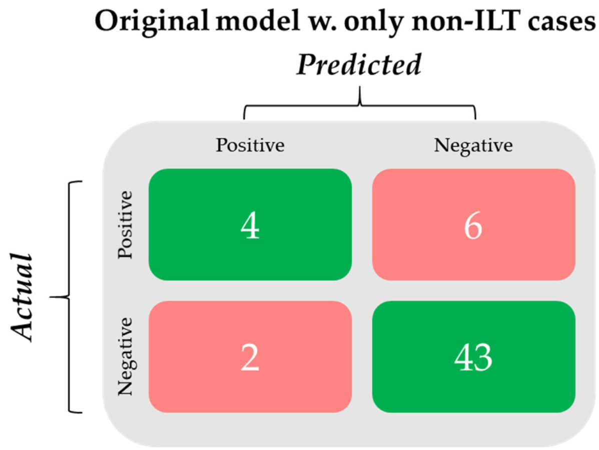

Figure 3. *Confusion matrix demonstrating the predictive performance of the original prediction model excluding cases with ILTs.*
